# Arabidopsis Glutathione-S-Transferases *GSTF11* and *GSTU20* Function in Aliphatic Glucosinolate Biosynthesis

**DOI:** 10.3389/fpls.2021.816233

**Published:** 2022-01-25

**Authors:** Aiqin Zhang, Rui Luo, Jiawen Li, Rongqing Miao, Hui An, Xiufeng Yan, Qiuying Pang

**Affiliations:** ^1^Key Laboratory of Saline-Alkali Vegetation Ecology Restoration (Northeast Forestry University), Ministry of Education, Harbin, China; ^2^College of Life Sciences and Food Engineering, Inner Mongolia Minzu University, Tongliao, China; ^3^Zhejiang Provincial Key Laboratory for Water Environment and Marine Biological Resources Protection, College of Life and Environmental Science, Wenzhou University, Wenzhou, China

**Keywords:** Arabidopsis, aliphatic glucosinolate, glutathione S-transferase, *GSTF11*, *GSTU20*

## Abstract

Glutathione (GSH) conjugation with intermediates is required for the biosynthesis of glucosinolate (GSL) by serving as a sulfur supply. Glutathione-S-transferases (GSTs) primarily work on GSH conjugation, suggesting their involvement in GSL metabolism. Although several GSTs, including GSTF11 and GSTU20, have been recently postulated to act in GSL biosynthesis, molecular evidence is lacking. Here, we demonstrated that *GSTF11* and *GSTU20* play non-redundant, although partially overlapping, roles in aliphatic GSL biosynthesis. In addition, *GSTU20* plays a more important role than *GSTF11*, which is manifested by the greater loss of aliphatic GSLs associated with *GSTU20* mutant and a greater number of differentially expressed genes in *GSTU20* mutant compared to *GSTF11* mutant. Moreover, a double mutation leads to a greater aggregate loss of aliphatic GSLs, suggesting that *GSTU20* and *GSTF11* may function in GSL biosynthesis in a dosage-dependent manner. Together, our results provide direct evidence that *GSTU20* and *GSTF11* are critically involved in aliphatic GSL biosynthesis, filling the knowledge gap that has been speculated in recent decades.

## Introduction

Glucosinolates (GSLs) are sulfur-rich secondary metabolites primarily present in *Brassicale* plants and well-known as important defense compounds that are beneficial to human health (Petersen et al., 2018). GSLs share a common core structure with an S-β-D-glucopyranose connected to an *O*-sulfated (Z)- thiohydroximate ester via a sulfur atom and are originally derived from amino acids (Agerbirk and Olsen, 2012; Blažević et al., 2020). Depending on the precursor amino acid, GSLs are grouped into three categories, including aliphatic GSLs (derived from alanine, isoleucine, leucine, methionine, and valine), indolic GSLs (derived from tryptophan) and aromatic GSLs (derived from phenylalanine and tyrosine) (Fahey et al., 2001; Halkier and Gershenzon, 2006).

The GSL biosynthetic pathway has been almost completely elucidated in recent decades (Halkier and Gershenzon, 2006; Sønderby et al., 2010; Nguyen et al., 2020). In brief, the biosynthesis of GSLs involves three key steps: side chain elongation with precursor amino acids, construction of a GSL core structure including sulfate assimilation, and secondary modifications of the side chain (Grubb and Abel, 2006; Sønderby et al., 2010; Petersen et al., 2018). The elongation process initiates with a transamination reaction catalyzed by branched-chain amino acid aminotransferases (BCATs). The side chain is then subjected to condensation with acetyl-CoA by methylthioalkylmalate synthases (MAMs), followed by isomerization and oxidative decarboxylation by isopropylmate isomerases (IPMs) and isopropylmalate dehydrogenases (IMDHs) (Kliebenstein et al., 2001; Schuster et al., 2006; Textor et al., 2007; He et al., 2009, 2011; Kroymann, 2011). Later, several biochemical reactions facilitate the production of the GSL core structure: oxidation by cytochrome P450 monooxygenases (cytochrome P450) of the CYP79 family, oxidation with conjugation by the CYP83 family, C-S cleavage by C-S lyase SUR1, glucosylation by glucosyltransferases of the UGT74 family and sulfation by sulfotransferases (SOT) (Bak and Feyereisen, 2001; Grubb et al., 2004; Mikkelsen et al., 2004; Piotrowski et al., 2004; Sønderby et al., 2010; Harun et al., 2020). Ultimately, the secondary modification of side chains undergoes oxidation, elimination, alkylation or esterification according to the distinct categories of GSLs (Harun et al., 2020).

As multifunctional enzymes, glutathione-S-transferases (GSTs) are primarily involved in the conjugation of the tripeptide glutathione (GSH) to the electrophilic center of lipophilic compounds (Labrou et al., 2015). Based on the similarity of amino acid sequences, plant specific GSTs are classified into the tau (GSTU) and phi (GSTF) types (Wagner et al., 2002). GSH contributes to the core structure synthesis of GSLs as a sulfur donor, raising the probability that GSTs are involved in the biosynthesis of GSLs. Indeed, GSTF9, GSTF10, and GSTU13 have been recognized to participate in the indolic GSLs biosynthesis (Piślewska-Bednarek et al., 2018). In contrast, it remains unclear that which GSTs function in aliphatic GSL biosynthesis, although GSTF11 and GSTU20 (Supplementary Figure 1) have been identified through multifaceted gene co-expression network analysis (Hirai et al., 2005, 2007; Wentzell et al., 2007; Czerniawski and Bednarek, 2018). The assumption of GSTF11 and GSTU20 are involved in GSL biosynthesis relies completely on *in silico* prediction, and molecular evidence is lacking.

In this study, we created *GSTF11* and *GSTU20* mutants using the CRISPR/Cas9 approach to ascertain the biological roles of *GSTF11* and *GSTU20* in GSL biosynthesis. Our results demonstrate that *GSTF11* and *GSTU20* are involved in aliphatic GSL biosynthesis with partially overlapping but non-redundant functions. Moreover, the aggregate loss of aliphatic GSLs observed in the double mutant implies that *GSTF11* and *GSTU20* also work in a dose-dependent manner. However, a substantial amount of aliphatic GSLs remain presence in the double mutant, suggesting that other GST family proteins are involved in aliphatic GSL biosynthesis, which awaits further exploration.

## Materials and Methods

### Plant Materials and Growth Conditions

The Columbia accession of *Arabidopsis thaliana* was used as the wild-type plant. Seeds were surface-sterilized and germinated on 1/2 Murashige and Skoog medium containing 2% sucrose and 0.8% Phytagar and grown in a 22°C growth chamber with a 16-h light and 8-h dark photoperiod after vernalization at 4°C for 3 days. One-week-old seedlings were then transferred to soil and grown under the aforementioned conditions.

To generate knockout mutants of GSTF11 (AT3G03190) and GSTU20 (AT1G78370) based on the CRISPR-Cas9 system, the online website CRISPR-PLANT was used to design the gRNA spacer sequences (Xie et al., 2014), and the CRISPR/Cas9 vector was constructed as described previously (Wang et al., 2015). The full-length CDS of GSTF11/U20 was amplified and then integrated into pDONR222 (entry vector) and pGWB551 (destination vector) to generate overexpression lines. For plant transformation, all binary vectors were transformed into *Agrobacteria* strain *GV3101* and subjected to the floral dipping method. Double mutants for GSTF11 and GSTU20 were created by crossing between *gstf11-2* and *gstu20-2* single mutants which use *gstf11-2* as pollen supplier. The primers used for constructs cloning and genotyping are listed in Supplementary Table 1.

### Co-expression Analysis

Glutathione-S-transferases and well-known aliphatic GSL synthesis genes (Supplementary Table 2) were retrieved from The Arabidopsis Information Resource (TAIR).^1^ Co-expression analysis was performed using ATTED 10.1^2^ and STRING^3^ platforms. Gene co-expression networks were drawn using the online tool NetworkDrawer.^4^

### Construction of β-Glucuronidase (GUS) Reporter and GUS Staining

*Arabidopsis* genomic DNA was extracted using the CTAB method and treated with RNase to remove RNA. For the generation of GUS reporter constructs, a genomic fragment of 668 and 1,454 bp upstream of translational start codon ATG of *GSTF11* and *GSTU20* was amplified, then cloned into pCAMBIA1305 using *Sal*I and *Nco*I restriction sites. The primers used in these experiments are listed in Supplementary Table 1. Histochemical GUS assays were performed on T3 generation of GUS stable expression lines by GUS staining solution (Solarbio) according to the instruction. The sample were firstly fixed in the fixation buffer for 45 min and washed by diluted GUS Buffer A with three times. Then immersed the tissues in 500 mL GUS staining buffer and incubated at 37°C for 24 h. The pictures of GUS expression in different tissues were captured by optical microscope (Olympus SZX10).

### Gene Expression Analysis

Total RNA was extracted from 100 mg leaf, stem and root of 3-week-old seedlings or flower and silique of mature plants with TRIzol reagent and then treated with gDNA Eraser to remove DNA contamination. cDNA was synthesized by Maxima H Minus reverse transcriptase (Thermo Scientific). SYBR Premix Ex TaqTM (Takara) was used for qRT-PCR analysis. The relative expression level of genes was calculated following the 2^–ΔΔCt^ method and normalized to the expression level of *ACTIN2*. Three technical replications were conducted for each biological experiment. The primers used in the qRT-PCR analysis are listed in Supplementary Table 1.

### Protoplast Isolation and Transfection

Two-week-old seedlings grown on 1/2 MS were used to isolate protoplasts as described previously with minor modifications (Jung et al., 2015), 10 g seedlings were used to generate more protoplast cells. The full-length coding sequences of GSTF11/U20 without stop codon was PCR amplified and cloned into pDONR222 vector by gateway BP Clonase (Invitrogen, 11789020), then inserted into the pSAT6-EGFP vector (Tzfira et al., 2005) by gateway LR Clonase (Invitrogen, 11791020). Approximately 10 μg DNA of recombined constructs (GSTF11/U20 fused with EGFP driven by 35S promoter) was transformed into protoplast cells as described by Yoo et al. (2007), incubated at room temperature for 16 h or longer, and observed for GFP signal under an Olympus BX53 microscope with a 40× objective to assess the subcellular localization of GSTF11/U20.

### Transcriptome Analysis

Total RNA was extracted from 3-week-old plants with TRIzol (Invitrogen) and purified using a GeneJET plant RNA purification kit (Thermo Fisher Scientific). RNA integrity and concentration were assessed by gel electrophoresis and using the Qubit^®^ 2.0 Fluorometer (Thermo Fisher Scientific). RNA (1.5 μg) was used for cDNA library preparation with the NEBNext^®^ Ultra™ RNA Library Prep Kit for Illumina^®^ (New England Biolabs, NEB) following the manufacturer’s protocol. Library quality was monitored using an Agilent Bioanalyzer 2100 (Agilent Technologies). The cDNA libraries were sequenced on an Illumina HiSeq 2500 platform, and 150-bp paired-end reads were generated.

Gene functional annotation was conducted by aligning reads to the Arabidopsis genome sequence (TAIR 10). Following alignment, the count of mapped reads from each sample was derived and normalized as RPKM (reads per kilobase of exon model per million mapped reads). Differentially expressed genes (DEGs) were identified using the DESeq R package (1.10.1). Genes with log2 fold change ≥ 1 and an FDR adjusted *p* value less than 0.05 were considered DEGs. GO term enrichment of DEGs was analyzed using tools in TAIR.

### Ultra-Performance Liquid Chromatography (UPLC) Analysis of Glucosinolates

Total GSLs were extracted from 150 mg leaves of 3-week-old seedlings or 20 mg mature seeds according to previously reported protocols (Chen et al., 2003; Alvarez et al., 2008). The sample were ground after adding 1 mL preheated 70% MeOH and vortex for 1 min, further incubated at 80°C for 10 min and centrifuge at 4,000 *g* for 10 min, collect supernatant and repeat the extraction one more time. Loading 1 mL DEAE-Sephadex A-25 (Sigma-Aldrich) into chromatographic column and cover with some quartz sand, add the extracted GSL sample into column then wash the column successively by 70% MeOH, ddH_2_O and 20 mM acetate solution, incubated the sample overnight at RT after adding 0.5 mL sulfatase (Sigma-Aldrich), collect GSL extraction by 1.5 mL ddH_2_O washing for further analysis. Each component of GSLs was analyzed using ultra-performance liquid chromatography (Waters ACQUITY UPLC M-Class) with an Atlantis T3 C18 column (2.1 mm × 150 mm, 3 μm, Waters) based on UV detector. The flow rate was kept at 0.4 mL/min, the column temperature was maintained at 25°C and the injection volume was 5 μL. 0.1% Trifluoroacetic acid in water as eluent A and methanol as eluent B was set as the mobile phase. Gradient elution conditions were as follows: 0–7.6 min, 0–60% B; 7.6–8.2 min, 60–100% B; 8.2–8.8 min, 100% B; 8.8–9.6 min, 100–0% B). Ten μL of 5 mM desulfonated benzyl GSL were added in each sample as the internal standard, quantification was obtained according to integrative peak areas using known relative response factors at 229 nm. Data presented are the means of three biological repeats.

The abbreviations of each component of GSLs described as follows: 3MSOP, 3-methylsulfinylpropyl GSL; 3BOP, 3-benzoylpropyl GSL; 3OHP, 3-hydroxylpropyl GSL; 4MTP, 4-methylthiobutyl GSL; 4MSOB, 4-methylsulfinylbutyl GSL; 4BOB, 4-benzoylbuthyl GSL; 5MSOP, 5-methylsulphinylpentyl GSL; 5MTP, 5-methylthiopentyl GSL; 6MSOH, 6-methylsulphinylhexyl GSL; 6MTH, 6-methylthiohexyl GSL; 7MTH, 7-methylthiohepthyl GSL; 8MSOO, 8-methylsulphinyloctyl GSL; 8MTO, 8-methylthiooctyl GSL; I3M, indolyl-3-methyl GSL; 1MOI3M, 1-methoxyindol-3-ylmethyl GSL; 4MOI3M, 4-methoxyindol-3-ylmethyl GSL; 4OHI3M, 4-hydroxyindol-3-ylmethyl GSL.

### Statistical Analysis

All claims of statistical significance (*p* < 0.05) were assessed by two-way ANOVA (*p* < 0.05) with Tukey’s HSD *post hoc* test.

## Results

### *GSTF11* and *GSTU20* Are Co-expressed With Numerous Genes Involved in Aliphatic Glucosinolate Biosynthesis

**FIGURE 1:**
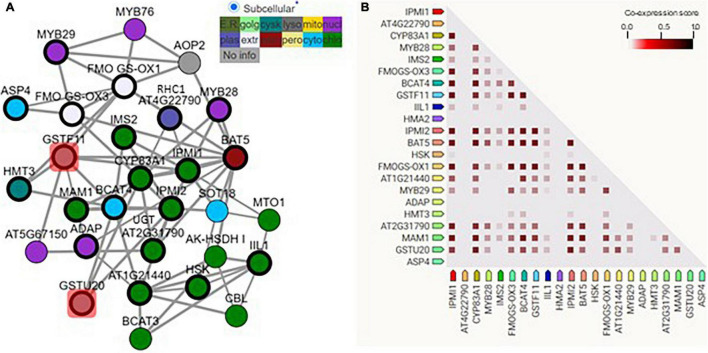
*GSTF11/U20* correlated with glucosinolate (GSL) biosynthesis genes. **(A)** The regulatory network of GSTF11/U20 with genes involved in the biosynthesis of aliphatic GSLs. GSTF11/U20 are highlighted by red squares connected with several detected aliphatic GSL genes. The genes present in the network image are listed in Supplementary Table 1. Different color nodes represent the predicted or experimental subcellular information of each gene. **(B)** Co-expression strength predicts the functional association between GSTF11/U20 and aliphatic GSL genes. The intensity of the color in the triangle matrices indicates the level of confidence that two proteins are functionally associated, and more confidence was noted when the score approached 1.

Many former gene co-expression analyses have identified *GSTF11* and *GSTU20* as candidate genes involved in the biosynthesis of aliphatic GSLs (Hirai et al., 2005, 2007; Wentzell et al., 2007; Hirai, 2009). To better interpret the co-expression, we constructed a gene regulatory network (GRN) of *GSTF11* and *GSTU20* with a total of 20 well-characterized genes involved in aliphatic GSL biosynthesis using the ATTED-II platform (Obayashi et al., 2018; Supplementary Table 2). As shown in Figure 1A, although all of the genes tested could be classified into a complex GRN module, a distinct but direct connection of genes with *GSTF11* or *GSTU20* was observed. In addition, the solid connections of *GSTF11* and *GSTU20* with a few known genes involved in aliphatic GSLs were further visualized after performing gene co-expression analysis using STRING co-expression viewers (Szklarczyk et al., 2019; Figure 1B). Moreover, GSTF11 and GSTU20 also displayed intimate correlations with aliphatic GSL biosynthetic genes in the protein-protein interaction network generated using the STRING program (Supplementary Figure 2). Overall, the integrative multiple *in silico* analyses support the putative involvement of *GSTF11* and *GSTU20* in aliphatic GSL biosynthesis.

### *GSTF11* and *GSTU20* Localize to the Cytosol and Display Distinct Tissue-Specific Expression Patterns

**FIGURE 2:**
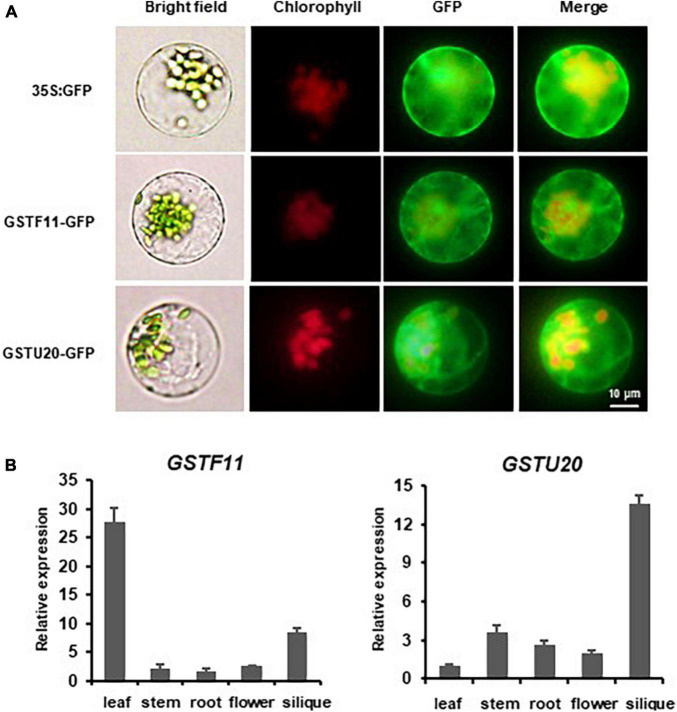
The expression pattern of *GSTF11*/*U20* in *Arabidopsis*. **(A)** Subcellular localization analysis of GSTF11/U20. Fluorescent signals of GSTF11-GFP and GSTU20-GFP fusion protein expressed in protoplasts of *Arabidopsis*. Green denotes the GFP signal, and red indicates the chlorophyll signal. **(B)** *GSTF11*/*U20* expression analysis by qPCR in different tissues. The tissue with the lowest expression was considered the standard and used to quantify the relative expression.

To gain insights into the subcellular localization of GSTF11 and GSTU20, the full-length coding sequences of *GSTF11* and *GSTU20* fused to enhanced green fluorescent protein (EGFP) under the control of 35S promoter were transformed into *Arabidopsis* protoplast cells. Using fluorescence microscopy, both GSTF11 and GSTU20 were observed primarily in the cytoplasm (Figure 2A).

The spatiotemporal expression patterns of *GSTF11* and *GSTU20* were first investigated by performing quantitative RT-PCR analyses. The results showed that *GSTF11* was highly expressed in rosette leaves, moderately expressed in siliques, and weakly expressed in stems, roots and flowers (Figure 2B). Compared with *GSTF11*, *GSTU20* seemed to be expressed in a complementary pattern, which was highly expressed in siliques but weakly expressed in leaves (Figure 2B). The tissue-specific expression patterns of *GSTF11* and *GSTU20* were also analyzed by generating transgenic Arabidopsis plants carrying GUS (β-glucuronidase) as a reporter gene driven by the native promoter of *GSTF11* and *GSTU20* for histochemical analysis. Consistent with the RT-PCR results, intense GUS staining of the *GSTF11* promoter was observed in leaves and siliques, but *GSTU20* promoter activity was mainly detected in siliques (Supplementary Figure 3). Overall, these results indicate that although the subcellular localization is the same, *GSTF11* and *GSTU20* exhibit distinct tissue- and organ-specific expression patterns in *Arabidopsis*.

### *GSTF11* and *GSTU20* Deficiencies Substantially Affect Aliphatic Glucosinolate Profiles

**FIGURE 3:**
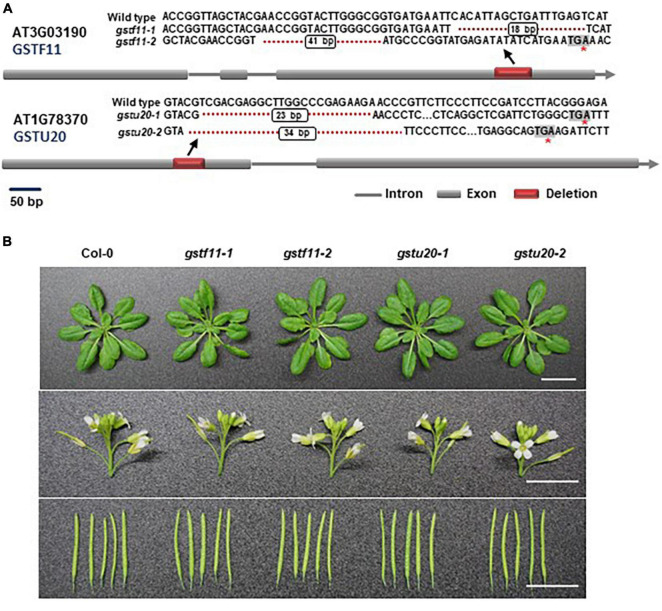
Morphological phenotypes of *gstf11* and *gstu20* mutants. **(A)** Diagram of mutations in *GSTF11/U20* genes generated by CRISPR-Cas9. Exons are represented by filled boxes, and introns are noted by lines. The red box indicates the deletion region in *GSTF11/U20*, and the red asterisk indicates a premature stop codon. **(B)** Rosette leaves, inflorescences and siliques of *gstf11* and *gstu20* mutants compare to wild type Col-0. Scale bars = 1 cm.

To ascertain the biological roles of *GSTF11* and *GSTU20* in GSL biosynthesis, we knocked out *GSTF11* and *GSTU20* using the CRISPR/Cas9 technique to obtain two mutant alleles for each gene. The *gstf11-1* and *gstf11-2* mutants contain an 18- or 41-bp deletion in the third exon of *GSTF11*, respectively. The deletion in *gstf11-1* begin the 486th base result in a frameshift mutation. The mutation in *gstf11-2* lead to the presence of a premature stop codon in the third exon (Figure 3A). Two GSTU20 mutations, *gstu20-1* and *gstu20-2*, contain a 23- or 34-bp deletion in the first exon respectively, both of which lead to the presence of a premature stop codon in the first exon (Figure 3A). The *gstf11* and *gstu20* mutants generated in this study exhibited no obvious morphological phenotypes (Figure 3B), indicating that neither *GSTF11* nor *GSTU20* is critically required for plant growth and development.

**FIGURE 4:**
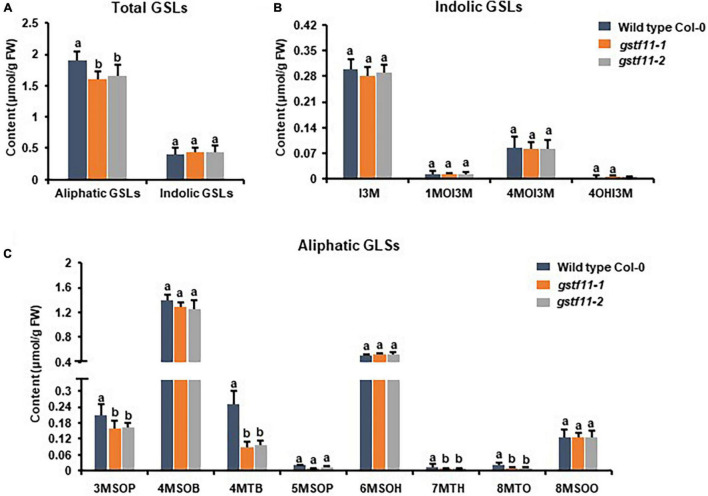
Glucosinolates content in the leaves of *gstf11* mutant lines. **(A)** Total aliphatic and indolic GSLs concentration in leaves from Col-0 and *gstf11* mutant lines. **(B)** Quantification of each component of aliphatic GSLs in *gstf11* mutant. **(C)** Quantification of each component of indolic GSLs in *gstf11* mutant. Values were obtained from three biological repeats. Letters indicate significant differences between wild type Col-0 and *gstf11* mutant lines as determined by two-way ANOVA (*p* < 0.05) with Tukey HSD *post hoc* test.

**FIGURE 5:**
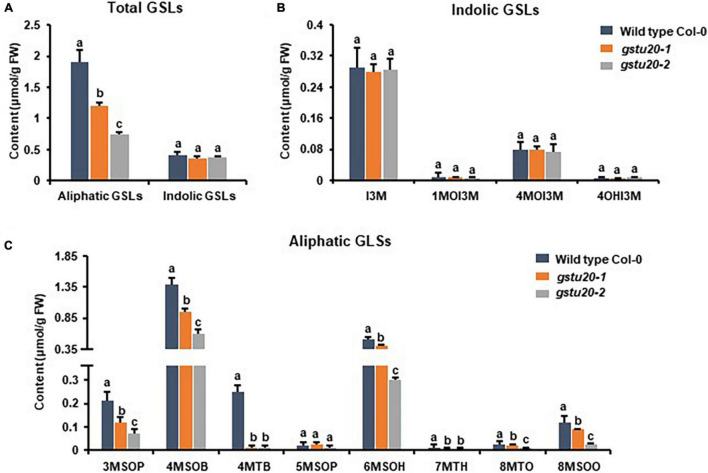
Glucosinolates content in the leaves of *gstu20* mutant lines. **(A)** Total aliphatic and indolic GSLs concentration in leaves from Col-0 and *gstu20* mutant lines. **(B)** Quantification of each component of aliphatic GSLs in *gstu20* mutant. **(C)** Quantification of each component of indolic GSLs in *gstu20* mutant. Values were obtained from three biological repeats. Letters indicate significant differences between wild type Col-0 and *gstu20* mutant lines as determined by two-way ANOVA (*p* < 0.05) with Tukey HSD *post hoc* test.

**FIGURE 6:**
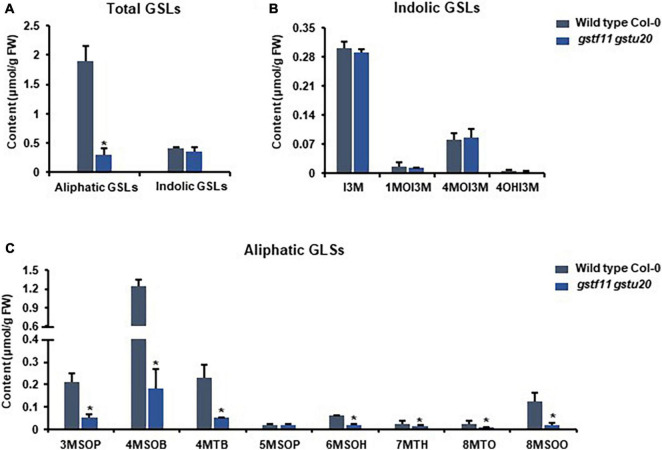
Glucosinolates accumulation in *gstf11gstu20* double mutant. **(A)** Total aliphatic and indolic GSLs concentration in leaves from Col-0 and *gstf11gstu20* mutant. **(B)** Quantification of each component of aliphatic GSLs in Col-0 and *gst* mutants. **(C)** Quantification of each component of indolic GSLs in Col-0 and *gst* mutants. Values were obtained from three biological repeats. Asterisks indicate significant differences between wild type Col-0 and *gst* mutants as determined by one-way ANOVA (*p* < 0.05) with Student’s *t*-test.

To ascertain the roles of *GSTF11* and *GSTU20* in GSL biosynthesis, GSL profiles were determined in the mutants described above (Supplementary Figure 4). Compared to wild-type plants, both *gstf11* and *gstu20* mutants exhibited substantial reduction in almost all categories of aliphatic GSLs with different side chain lengths in both 3-week-old leaves (Figures 4, 5). In contrast, no significant changes in the abundance of indolic GSLs were noted in *gstf11* (Figures 4A,B) and *gstu20* (Figures 5A,B) mutants, suggesting that *GSTF11* and *GSTU20* are not required for indolic GSL biosynthesis. For aliphatic GSLs, the formation of 3MSOP (3-methylsulfinylpentyl GSL), 4MTB (4-methylthiobutyl GSL), 7MTH (7-methylsulfinylheptyl GSL), and 8MTO (8-methylthiooctyl GSL) were significantly affected by GSTF11 deficiency (Figure 4C). In the leaves of *gstu20* mutant plants, the accumulation of aliphatic GSLs exhibited a striking defect in both mutant alleles, which was more severe in *gstu20-2* than *gstu20-1* (Figure 5C). Except 5MSOP (5-methylsulphinylpentyl glucosinolate), the accumulation of all types aliphatic GSLs were reduced in *gstu20* mutant, and *gstu20-2* accumulated less these GSLs than *gstu20-1* that is more likely a weak allele (Figure 5C). To sum up, the content of aliphatic GSLs with 3C, 4C, 7C, and 8C side chains were both affected by *GSTF11* and *GSTU20* mutation, and the accumulation of 6C aliphatic GSL only changed in *gstu20* mutant (Supplementary Figure 5A). To test whether GSTF11 and GSTU20 also play synergistic functions in GSL biosynthesis, we constructed *gstf11* and *gstu20* double mutant (Supplementary Figure 6). Interestingly, compared to the two single mutants, the reduction in aliphatic GSLs was further exaggerated in the double mutant (Figures 6A,C). Like the measurement in single mutants, there is no significant change of indolic GSLs content in *gstf11gstu20* double mutant (Figures 6A,B).

**FIGURE 7:**
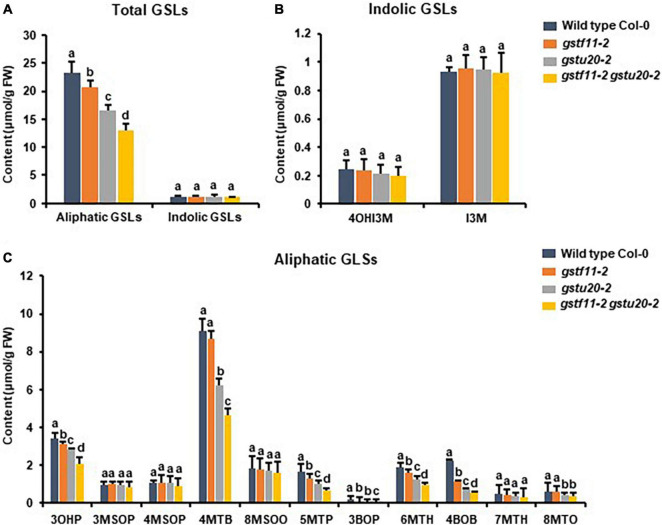
Glucosinolates content in the seeds of *gst* mutants. **(A)** Total aliphatic and indolic GSLs concentration in seeds from Col-0 and *gst* mutants. **(B)** Quantification of each component of aliphatic GSLs in *gst* mutants. **(C)** Quantification of each component of indolic GSLs in *gst* mutants. Values were obtained from three biological repeats. Letters indicate significant differences between wild type Col-0 and *gst* mutant lines as determined by two-way ANOVA (*p* < 0.05) with Tukey HSD *post hoc* test.

To further determine the correlation between the tissue specific-expression pattern of *GSTs* and GSL biosynthesis, we also detected the GSLs content in mature seeds of *gstf11-2* and *gstu20-2* mutants. As shown, the mutation of *GSTF11* and *GSTU20* significantly affected total content of aliphatic GSLs but not indolic GSLs in the seeds, and more aliphatic GSLs were lost in *gstf20* (Figures 7A,B). Among aliphatic GSL components, the accumulation of 3OHP (3-hydroxylpropyl GSL), 3BOP (3-benzoylpropyl GSL), 4BOB (4-benzoylbuthyl GSL), 5MTP (5-methylthiopentyl GSL), and 6MTH (6-methylthiohexyl GSL) decreased in *gstf11* and *gstf20*, and most of them were lost more in *gstf20*, but we found the content of 4MTB and 8MTO in seeds were only affected by *GSTU20* mutation (Figure 7C). The results showed that GSTF11 and GSTU20 regulate the accumulation of aliphatic GSLs in both leaves and seeds, which is not directly correlated with the gene expression abundance in the particular tissues (Figure 2B), it is more likely GSTU20 play a primary role in the biosynthesis of aliphatic GSLs no matter the transcript level high or low in seed and leaf. Similar with the accumulation in leaves, the aliphatic GSLs content in *gstf11 gstf20* was lower than single mutants (Figure 6C), also showed a synergistic effect between *GSTF11* and *GSTU20*.

These results indicated that both *GSTF11* and *GSTU20* are non-redundantly involved in aliphatic GSL biosynthesis, GSTF11 and GSTU20 act on aliphatic GSL biosynthesis in a dosage-dependent manner. Moreover, the reduction in aliphatic GSLs was more severe in *gstu20* compared to *gstf11* mutant. Three components of aliphatic GSLs like 4MSOB (4-methylsulfinylbutyl glucosinolate), 6MSOH (6-methylsulphinylhexyl glucosinolate) and 8MSOO (8-methylsulphinyloctyl glucosinolate) reduced in *gstu20* mutant but maintained wild type level in *gsts11* leaves (Figures 4, 5), indicating that *GSTU20* plays a greater role in GSL biosynthesis than *GSTF11*.

### *GSTF11* and *GSTU20* Deficiencies Caused Partially Overlapping Transcriptome Alterations

**FIGURE 8:**
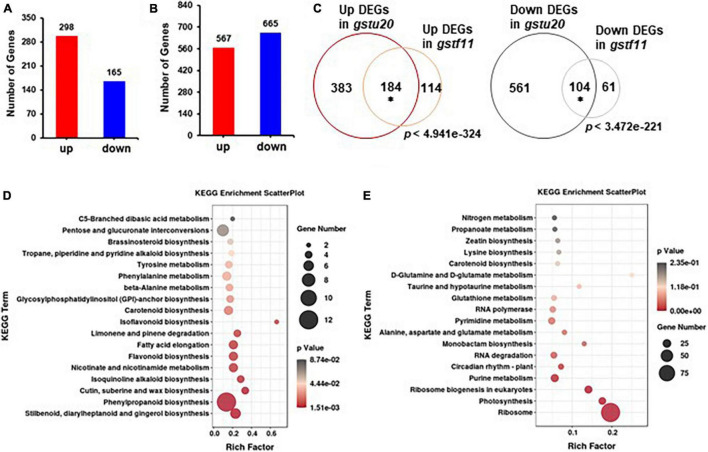
Gene expression profiles identified in *gst* mutant lines. The number of differentially expressed genes (DEGs) identified in *gstf11* **(A)** and *gstu20* **(B)** mutants. **(C)** Overlap of up- or downregulated genes between the *gstf11* and *gstu20* mutant. Venn diagrams were drawn in BioVenn (http://www.biovenn.nl/). Asterisks indicate the statistical significance (*p* < 0.01) of overlap, which was calculated using the online tool at http://nemates.org/MA/progs/overlap_stats.html. KEGG pathways were enriched for **(D)** up- and **(E)** downregulated DEGs expressed in the *gstu20* mutant. A plot diagram was drawn by ggplot2 (https://rdocumentation.org/packages/ggplot2/versions/2.1.0). The Rich factor indicates the number of identified DEGs versus the total genes involved in the metabolic pathways and describes the significance of pathway enrichment. The dot size represents the number of identified DEGs in each pathway and *p* value was indicated by a color bar.

To understand molecular changes in response to the perturbation of *GSTF11* and *GSTU20*, RNA-seq analysis was performed to examine the transcript profiles in both *gstf11-2* and *gstu20-2* mutant leaves with three biological replicates (Supplementary Table 3). By applying a false discovery rate (FDR) ≤ 0.05 and fold change ≥ 2, a total of 463 differentially expressed genes (DEGs) were identified, including 298 up- and 165 downregulated genes in *gstf11* compared to wild-type plants (Figure 8A and Supplementary Table 4). In *gstu20*, 1,232 genes were identified as DEGs, including 567 up- and 665 downregulated genes (Figure 8B and Supplementary Table 5). The larger number of DEGs observed in the *gstu20* mutant compared with the *gstf11* mutant suggests that defective *GSTU20* causes a greater extent of cellular response relative to *GSTF11*. These DEGs were enriched in various biological terms shown in Supplementary Figures 7, 8, the metabolic processes related to plant growth and development, stress response were also enriched, suggesting that GST mutation results in perturbation of multiple metabolic processes. More importantly, a large proportion of DEGs overlapped in the *gstf11* and *gstu20* mutants, accounting for 62% (*n* = 298) and 63% (*n* = 165) of the up- and downregulated DEGs in *gstf11*, respectively (Figure 8C).

As the majority of DEGs in *gstf11* were included in the *gstu20* mutant, we performed Gene Ontology (GO) enrichment analysis (Mi et al., 2019) using DEGs in *gstf20* as the representative mutant. The results showed that the upregulated DEGs were mainly enriched in response to stimulus, transcription factor activity, and metabolic processes (Supplementary Figure 7A and Supplementary Table 5). In contrast, the downregulated DEGs were primarily enriched in translation and rRNA processing (Supplementary Figure 7B and Supplementary Table 5). Moreover, KEGG pathway analysis (Kanehisa et al., 2016) revealed that upregulated DEGs were highly enriched in multiple primary and secondary metabolic processes (Figure 8D). Surprisingly, the downregulated DEGs were principally enriched in ribosome and ribosome biogenesis (Figure 8E). These results suggest that the perturbation of *GSTF11* and *GSTU20* caused a wide range of cellular alterations likely result from the disrupted GSL biosynthesis.

## Discussion

The formation of the GSL core structure requires an intermediate with a GSH conjugate serving as a sulfur supply. The enzymatic activity of GST family proteins in conjugating GSH into substantial metabolic intermediates has enabled researchers to postulate that some GST proteins are involved in GSL biosynthesis. Indeed, GSTF9, GSTF10, and GSTU13 have been recently identified to participate in indolic GSL biosynthesis (Piślewska-Bednarek et al., 2018). In contrast, which GSTs undertake a function in aliphatic GSL biosynthesis is merely conceptual and simply based on *in silico* transcriptional co-expression analysis (Wentzell et al., 2007; Bednarek et al., 2009; Geu-Flores et al., 2011; Klein and Sattely, 2017). In this study, we provide evidence *in planta* demonstrating that both of *GSTF11* and *GSTU20* are involved in aliphatic GSL biosynthesis with two unanticipated but intriguing features.

### *GSTU20* Plays a Greater Role in Aliphatic Glucosinolate Biosynthesis Than *GSTF11*

The greater loss of aliphatic GSLs in the loss-of-function of *gstu20* mutants compared to *gstf11* mutants in both leaves and seeds supports the notion that *GSTU20* plays a more important role than *GSTF11* in aliphatic GSL biosynthesis. This conclusion is further corroborated by the finding that more severe alteration of the transcriptome profile occurs in *gstu20* mutant compared with *gstf11* mutant. However, based on tissue-specific patterns, *GSTFU20* was expressed at a lower level relative to *GSTF11*, which seems contradictory to its superior function in comparison to *GSTF11*. Three possibilities might explain this phenomenon. First, the deletion in *gstf11* mutants happened at the last exon of *GSTF11*, which may generate a truncated protein and still remain function in GSL biosynthesis. Second, similar to other enzymes, the *in vivo* catalytic activity of GSTFU20 and GSTF11 is derived from proteins, the cellular content of which is determined by multiple steps of gene expression regulation, including transcription and posttranscription. In this context, the low level of GSTFU20 transcripts may be accompanied by a high extent of translational efficiency, leading to an increase in protein abundance. The main alternative possibility is that the enzymatic activity of distinct member of GSTs is variable and influenced by protein structure *in planta*. GSTU20 has been assayed for activity toward model xenobiotic substrate CDNB and BITC which are the typical GST substrates, revealing high GSH-conjugating activity (Gil and MacLeod, 1980; Edwards and Dixon, 2005; Dixon et al., 2009). The enzymatic activity of GSTF11 was undetectable because of the protein could not be isolated and purified *in vitro* due to the rare abundance. The metabolic engineering in tobacco and yeast indicated that the expression of GSTF11 is not essential for GSL production, even though it could increase GSL accumulation level (Mikkelsen et al., 2010, 2012). In this scenario, GSTU20 may have higher degree of GSH-conjugating activity than GSTF11. Regardless of which possibility is true, the levels of protein and its derived enzymatic activity should be validated in future studies.

### *GSTF11* and *GSTU20* Function in Aliphatic Glucosinolates Biosynthesis in a Dosage-Dependent Manner

In both the *gstf11* and *gftu20* mutants, the total abundance of aliphatic GSLs decreased, indicating that *GSTF11* and *GSTU20* are not redundant to each other. In addition, the altered pattern in terms of GSLs with different side chains was similar between the *gstf11* and *gftu20* mutants, suggesting that *GSTF11* and *GSTU20* functionally overlap. This overlap is further reflected by the fact that a large number of DEGs also overlapped in the *gstf11* and *gftu20* mutants. These characteristics of non-redundance and overlap seem mutually contradictory. However, this finding could be simply explained if we consider that both *GSTF11* and *GSTU20* work in aliphatic GSL biosynthesis in a dosage-dependent manner, and the loss of *GSTF11* or *GSTU20* could result in reduced GST activity required for the GSH-conjugation step. This assumption is further corroborated by the aggregate loss of aliphatic GSLs in the *gstf11 gftu20* double mutants. In addition, it is worth mentioning that the double mutation of *GSTF11* and *GSTU20* caused a dramatic decrease but did not completely abolish the formation of aliphatic GSLs, implying the existence of other isoforms like another unknown GST members that additionally functions in aliphatic GSL biosynthesis. Moreover, both GSTF11 and GSTU20 showed tissue-specific expression patterns but the content of GSLs was not intimately connected with the transcript abundance, enhancing the prospect that other GST family proteins also work on GSL biosynthesis in different tissues besides GSTF11 and GSTU20, maybe these GST members make distinct contribution on the accumulation of GSLs in the tissues including leaf, stem, root, flower and seed, which deserves further investigation in the future.

## Data Availability Statement

The data presented in the study are deposited in the DRYAD repository, accession number https://doi.org/10.5061/dryad.nvx0k6dtk.

## Author Contributions

QP and XY proposed the project and designed the experiments. AZ, RL, JL, RM, and HA performed the experiments and analyzed the data. AZ and QP prepared the manuscript with contributions from other authors. All authors contributed to the article and approved the submitted version.

## Conflict of Interest

The authors declare that the research was conducted in the absence of any commercial or financial relationships that could be construed as a potential conflict of interest.

## Publisher’s Note

All claims expressed in this article are solely those of the authors and do not necessarily represent those of their affiliated organizations, or those of the publisher, the editors and the reviewers. Any product that may be evaluated in this article, or claim that may be made by its manufacturer, is not guaranteed or endorsed by the publisher.

## Footnotes

1
https://www.arabidopsis.org/index.jsp

2
https://atted.jp/

3
https://string-db.org

4
https://atted.jp/top_draw/#CoexViewer
