# Supplementary material for: Arabidopsis Glutathione-S-Transferases GSTF11 and GSTU20 Function in Aliphatic Glucosinolate Biosynthesis
Source: Front Plant Sci. 2022 Jan 25;12:816233. doi: 10.3389/fpls.2021.816233 (PMC8821908; doi:10.3389/fpls.2021.816233)
Supplement: Supplementary file 1 [file Presentation_1.PPTX]

## Slide 1
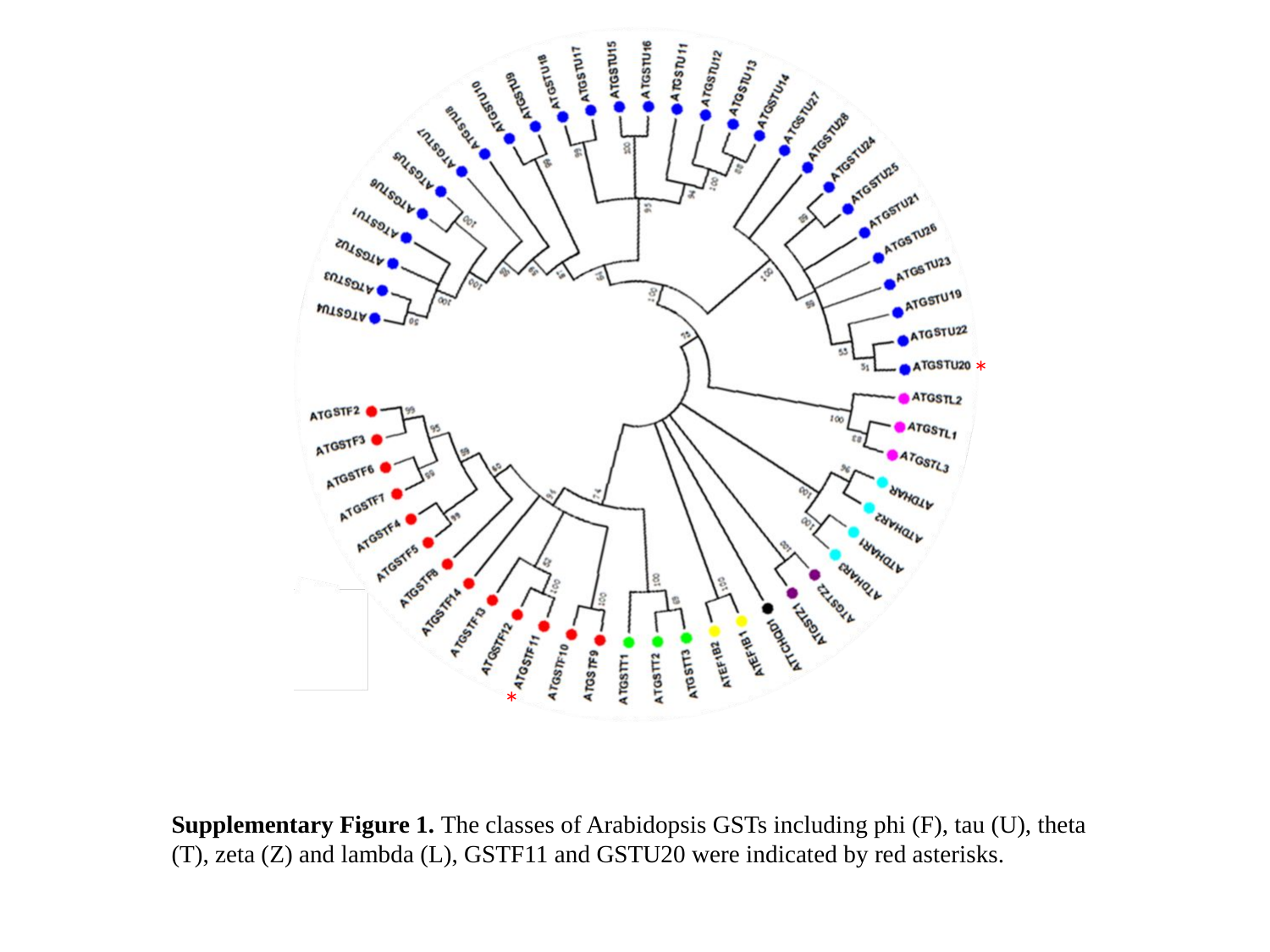

*
*
Supplementary Figure 1. The classes of Arabidopsis GSTs including phi (F), tau (U), theta (T), zeta (Z) and lambda (L), GSTF11 and GSTU20 were indicated by red asterisks.

## Slide 2
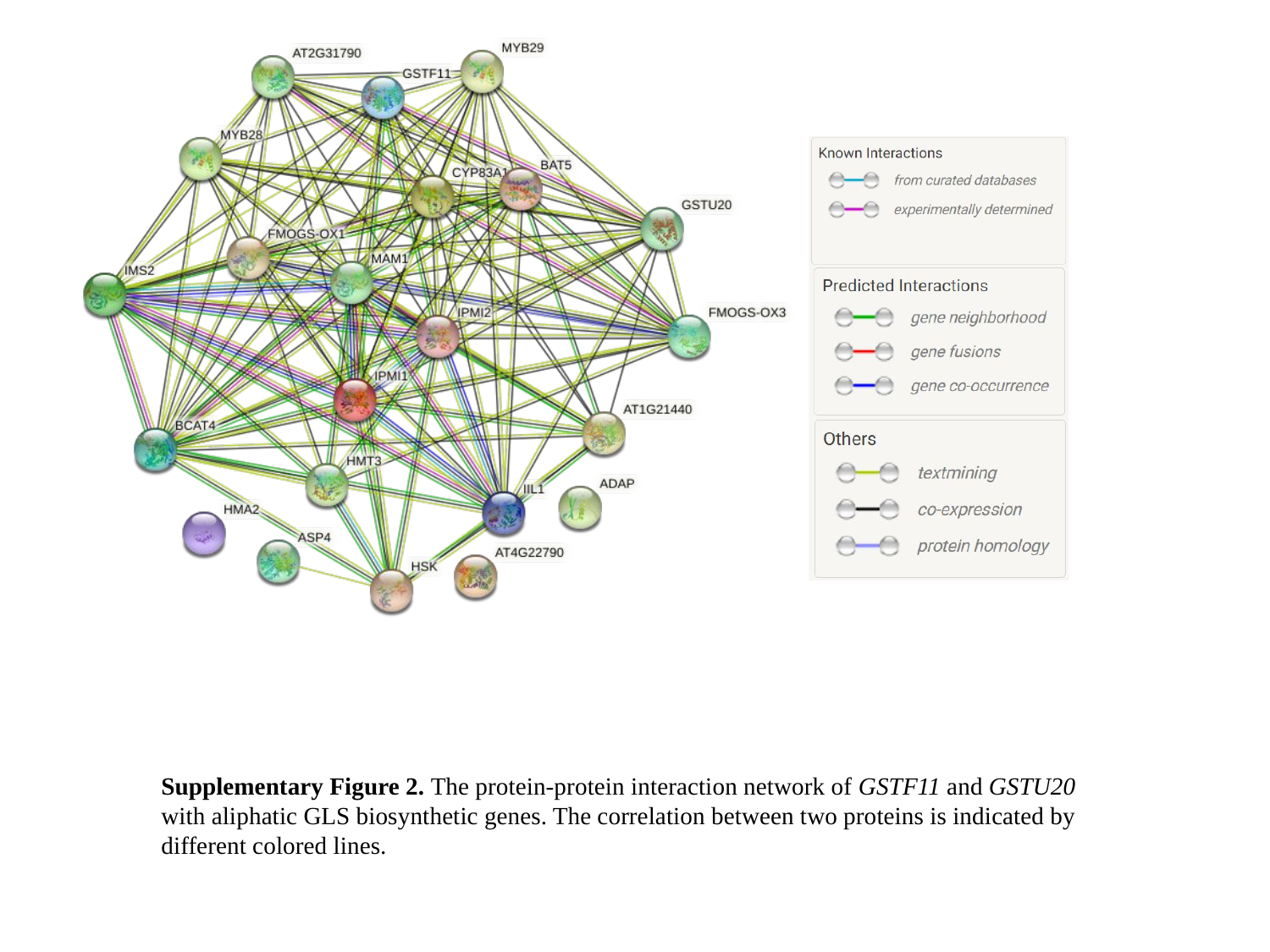

Supplementary Figure 2. The protein-protein interaction network of GSTF11 and GSTU20 with aliphatic GLS biosynthetic genes. The correlation between two proteins is indicated by different colored lines.

## Slide 3
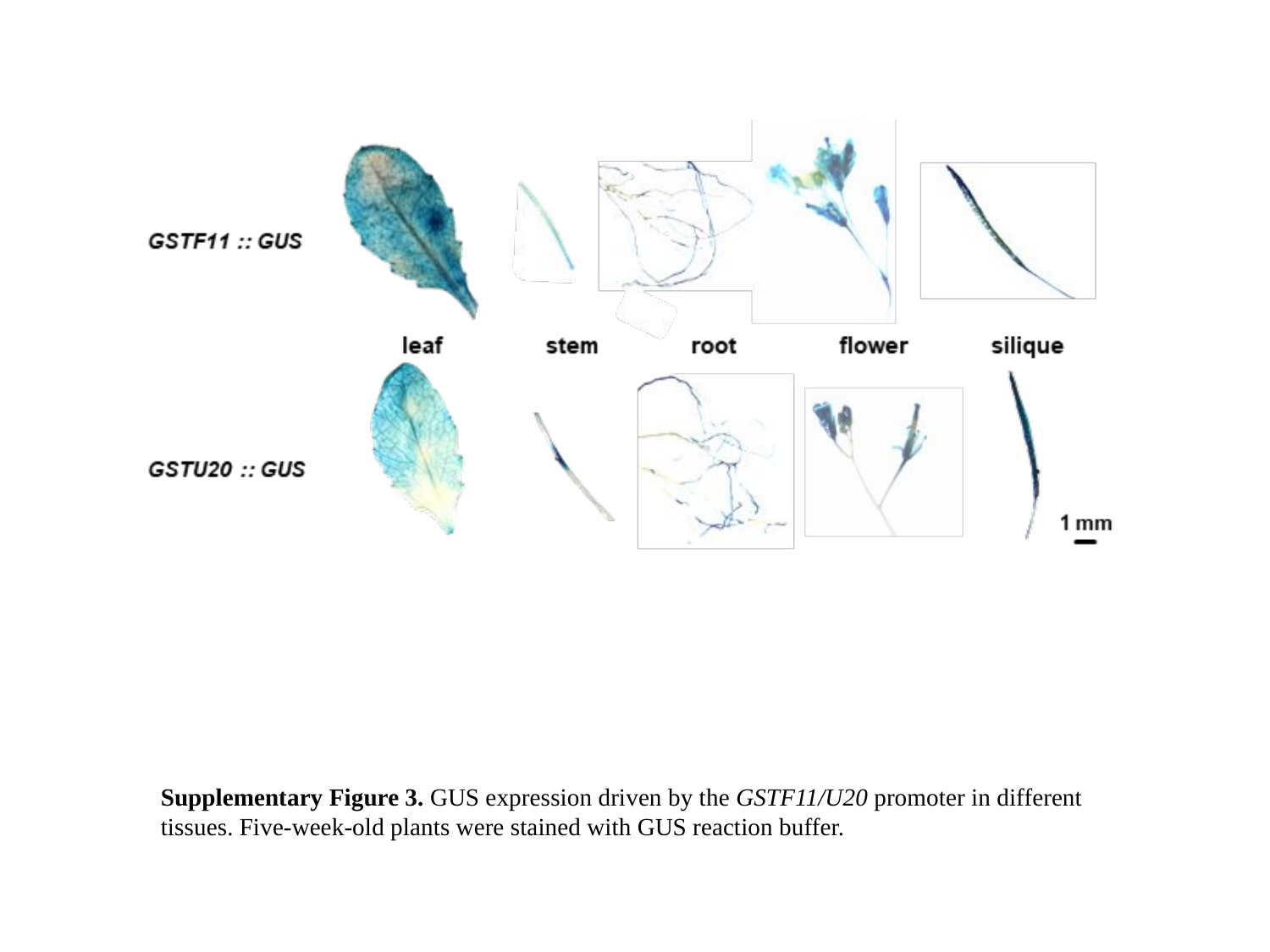

Supplementary Figure 3. GUS expression driven by the GSTF11/U20 promoter in different tissues. Five-week-old plants were stained with GUS reaction buffer.

## Slide 4
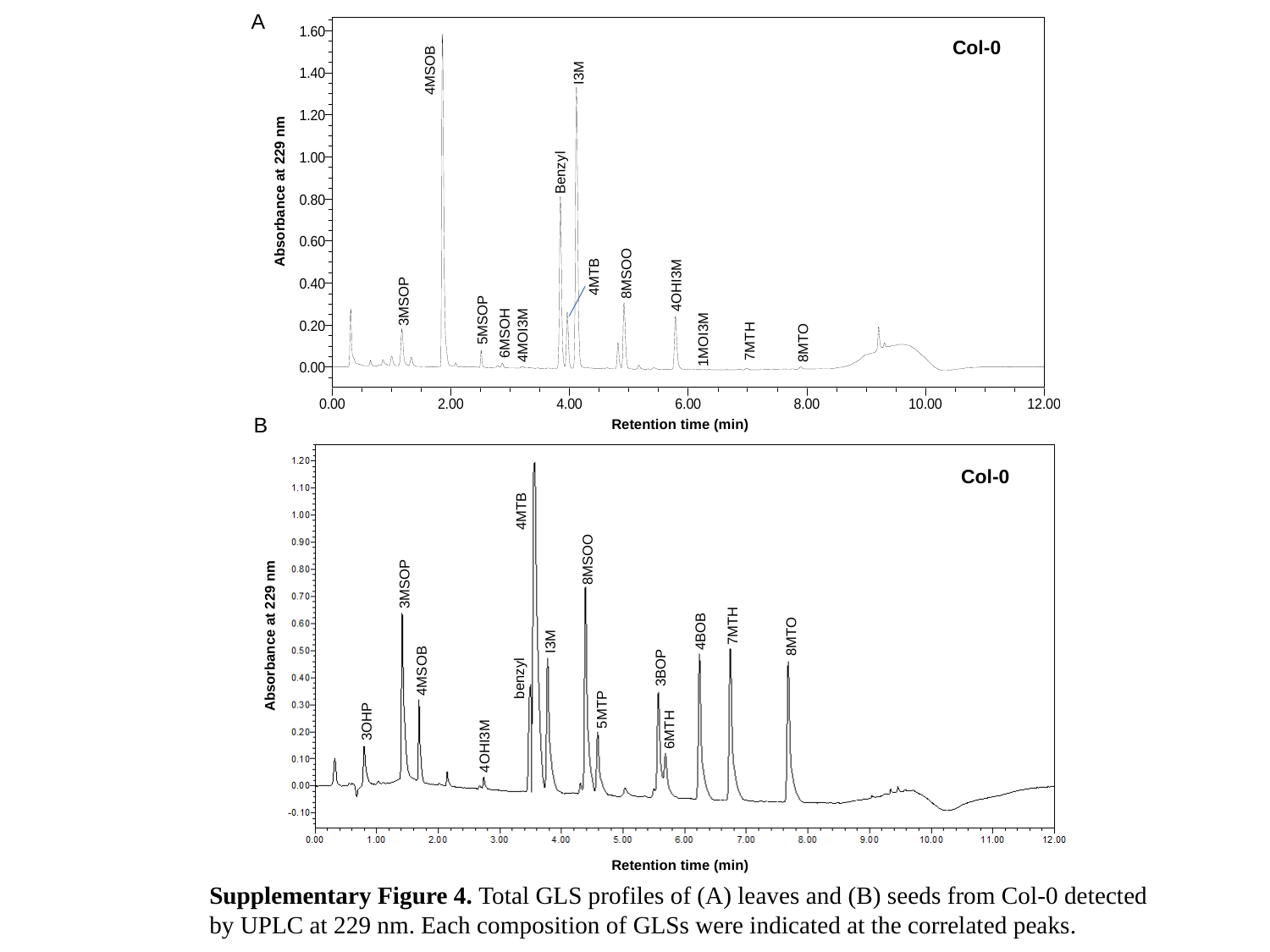

A
I3M
4MSOB
Benzyl
Absorbance at 229 nm
8MSOO
4MTB
4OHI3M
3MSOP
5MSOP
4MOI3M
6MSOH
1MOI3M
8MTO
7MTH
Retention time (min)
Col-0
B
Col-0
4MTB
8MSOO
3MSOP
7MTH
8MTO
I3M
4BOB
Absorbance at 229 nm
benzyl
3BOP
4MSOB
5MTP
6MTH
4OHI3M
3OHP
Retention time (min)
Supplementary Figure 4. Total GLS profiles of (A) leaves and (B) seeds from Col-0 detected by UPLC at 229 nm. Each composition of GLSs were indicated at the correlated peaks.

## Slide 5
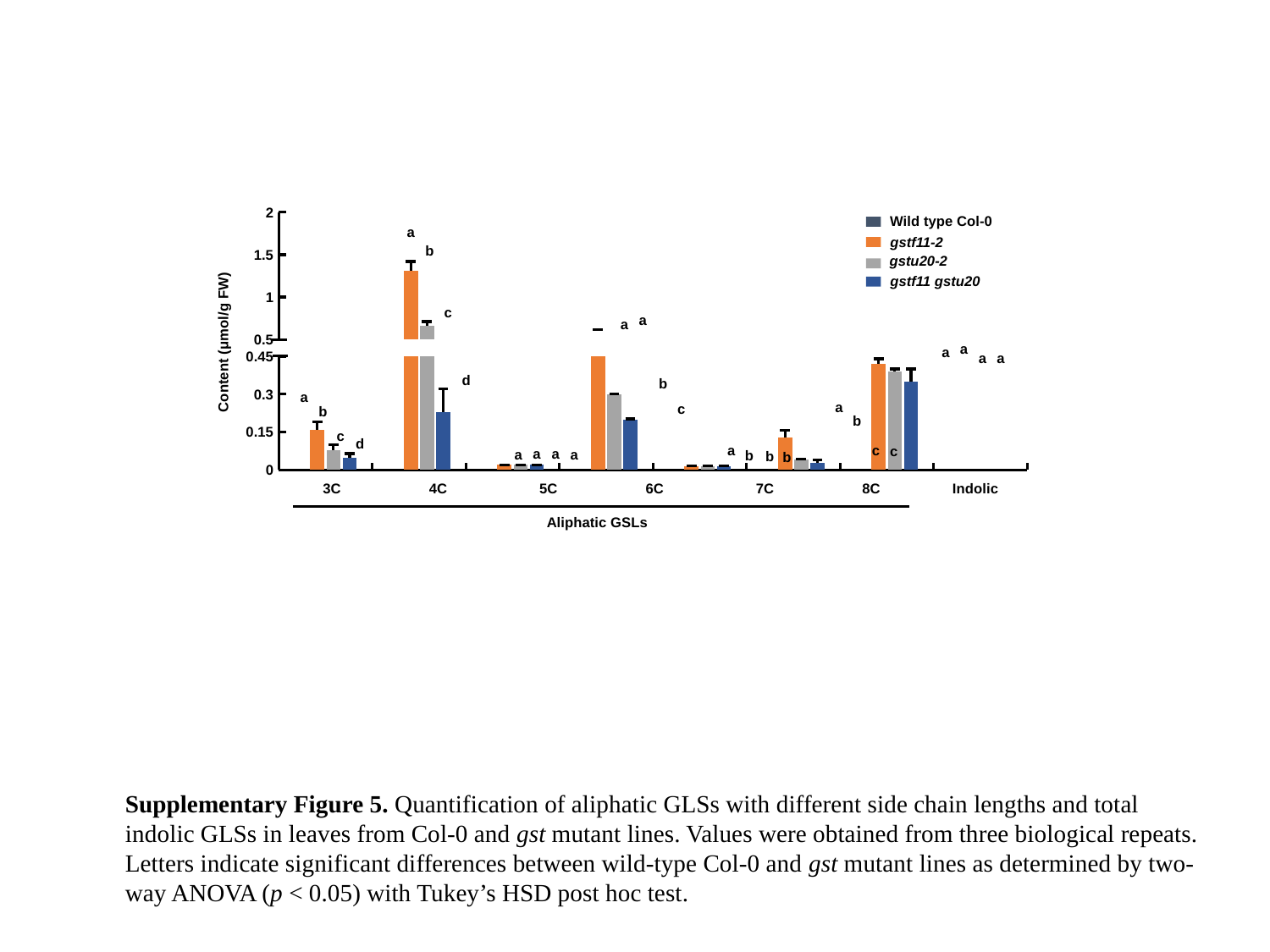

### Chart
| Category | | | | |
|---|---|---|---|---|Wild type Col-0
gstf11-2
gstu20-2
gstf11 gstu20
a
b
c
a
Content (μmol/g FW)
a
### Chart
| Category | | | | |
|---|---|---|---|---|
a
a
a
a
d
b
a
a
c
b
b
c
d
c
a
c
a
a
a
a
b
b
b
 3C 4C 5C 6C 7C 8C Indolic
Aliphatic GSLs
Supplementary Figure 5. Quantification of aliphatic GLSs with different side chain lengths and total indolic GLSs in leaves from Col-0 and gst mutant lines. Values were obtained from three biological repeats. Letters indicate significant differences between wild-type Col-0 and gst mutant lines as determined by two-way ANOVA (p < 0.05) with Tukey’s HSD post hoc test.

## Slide 6
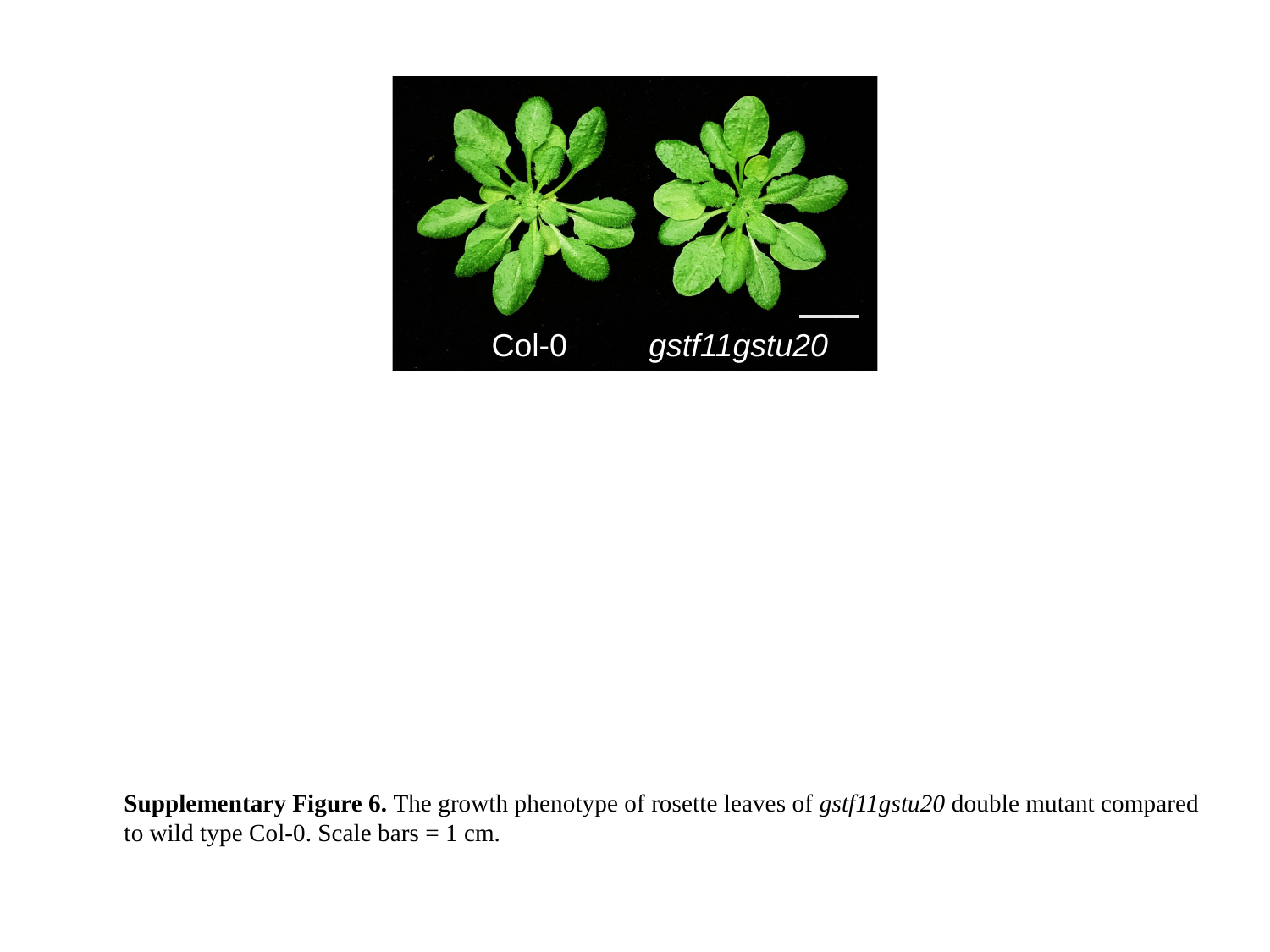

Col-0
gstf11gstu20
Supplementary Figure 6. The growth phenotype of rosette leaves of gstf11gstu20 double mutant compared to wild type Col-0. Scale bars = 1 cm.

## Slide 7
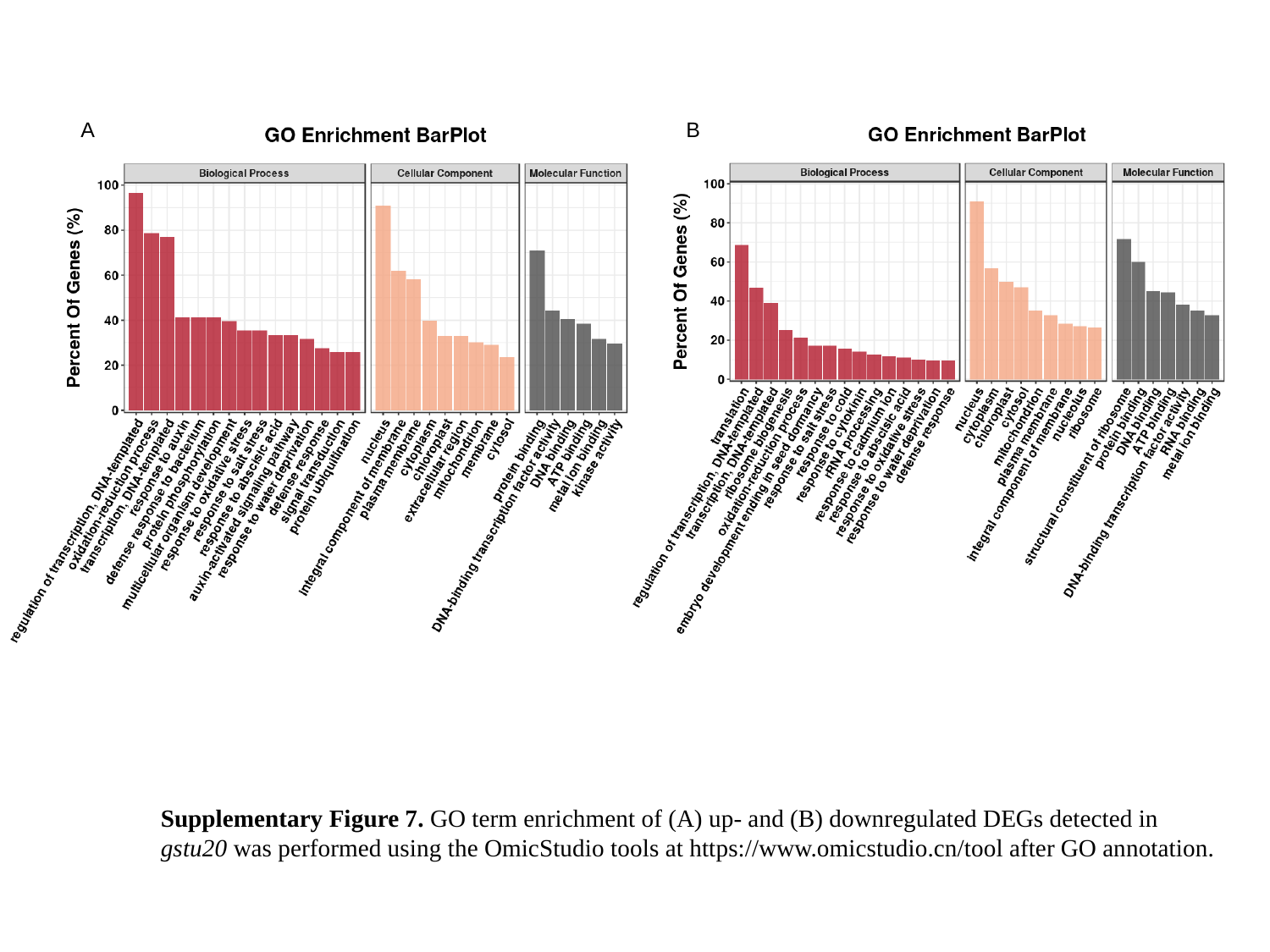

A
B
Supplementary Figure 7. GO term enrichment of (A) up- and (B) downregulated DEGs detected in gstu20 was performed using the OmicStudio tools at https://www.omicstudio.cn/tool after GO annotation.

## Slide 8
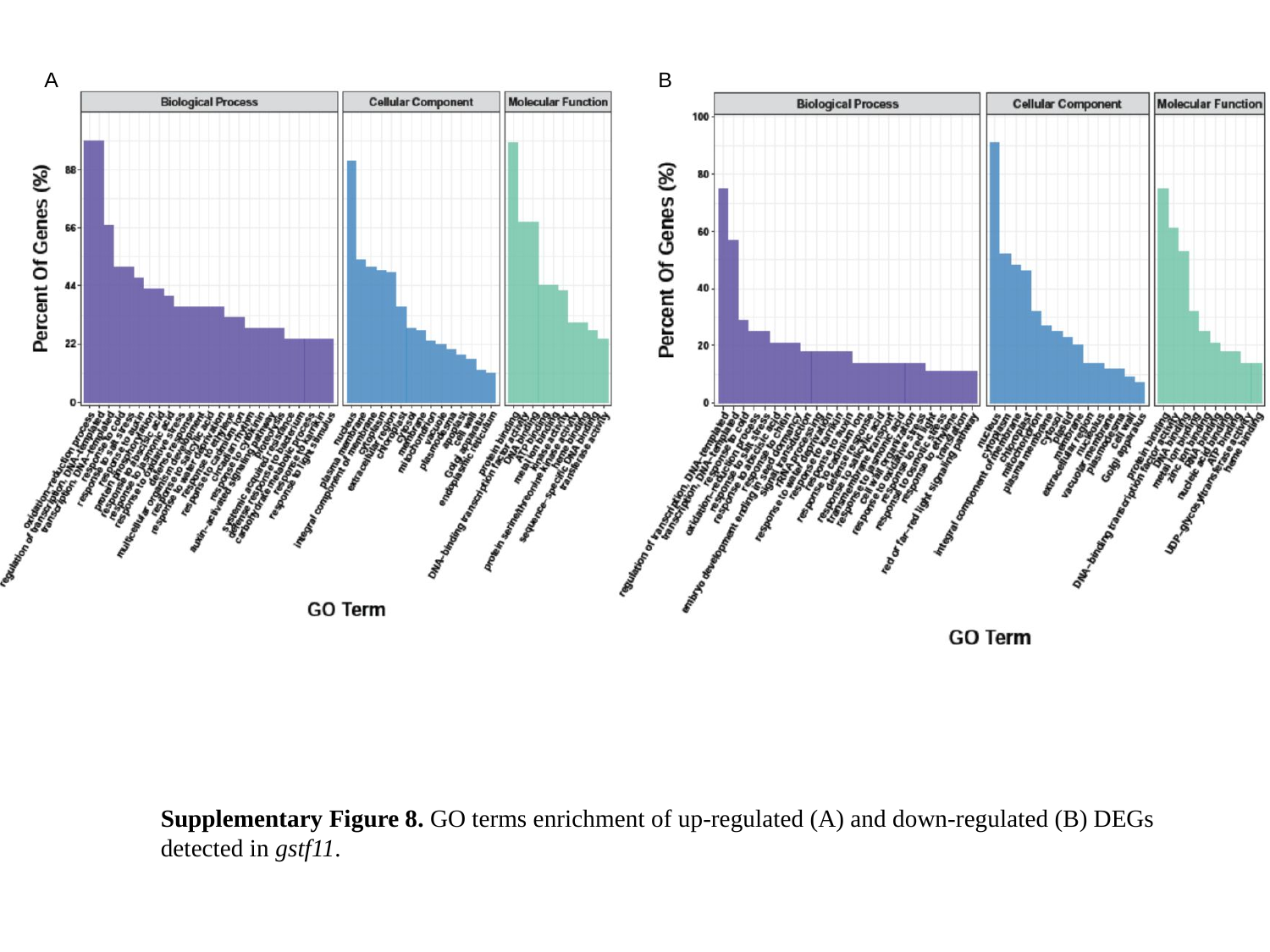

A
B
Supplementary Figure 8. GO terms enrichment of up-regulated (A) and down-regulated (B) DEGs detected in gstf11.

## Slide 9
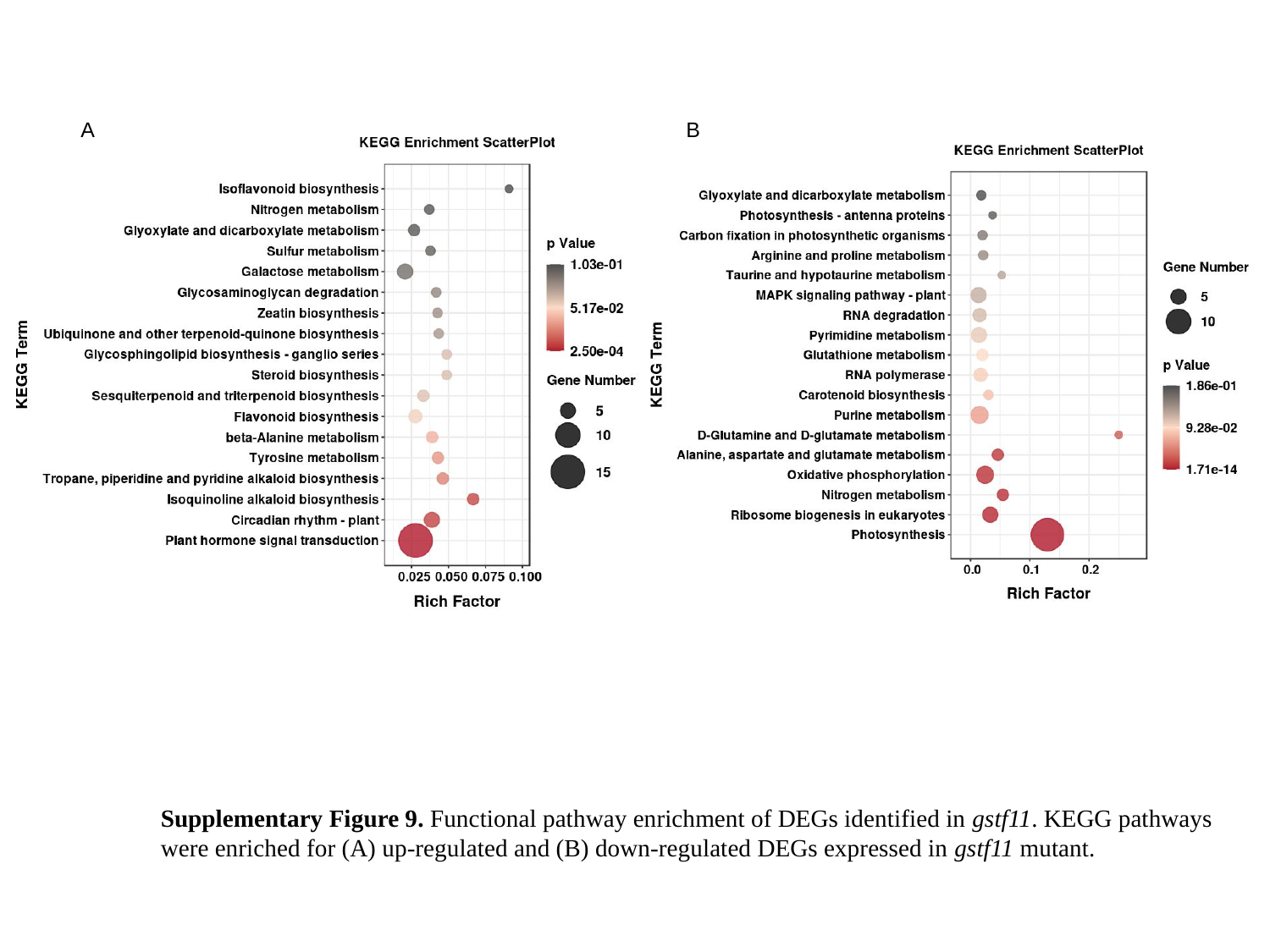

A
B
Supplementary Figure 9. Functional pathway enrichment of DEGs identified in gstf11. KEGG pathways were enriched for (A) up-regulated and (B) down-regulated DEGs expressed in gstf11 mutant.
